# Optimization and intelligent power management control for an autonomous hybrid wind turbine photovoltaic diesel generator with batteries

**DOI:** 10.1038/s41598-023-49067-4

**Published:** 2023-12-09

**Authors:** D. Rekioua, Z. Mokrani, K. Kakouche, T. Rekioua, A. Oubelaid, P. O. Logerais, Enas Ali, Mohit Bajaj, Milkias Berhanu, Sherif S. M. Ghoneim

**Affiliations:** 1 Laboratoire LTII, Faculté de Technologie, Université de Bejaia, 06000 Bejaïa, Algeria; 2 CERTES, IUT de Sénart-Fontainebleau, Univ. Paris-Est, Lieusaint, France; 3 Faculty of Engineering and Technology, Future University in Egypt, New Cairo, 11835 Egypt; 4 Department of Electrical Engineering, Graphic Era (Deemed to be University), Dehradun, 248002 India; 5 Graphic Era Hill University, Dehradun, 248002 India; 6 Applied Science Research Center, Applied Science Private University, Amman, 11937 Jordan; 7 Department of Electrical Power and Control Engineering, Adama Science and Technology University, Adama, Ethiopia; 8 Department of Electrical Engineering, College of Engineering, Taif University, P.O. Box 11099, 21944 Taif, Saudi Arabia

**Keywords:** Energy science and technology, Engineering, Mathematics and computing

## Abstract

In this paper, a critical issue related to power management control in autonomous hybrid systems is presented. Specifically, challenges in optimizing the performance of energy sources and backup systems are proposed, especially under conditions of heavy loads or low renewable energy output. The problem lies in the need for an efficient control mechanism that can enhance power availability while protecting and extending the lifespan of the various power sources in the system. Furthermore, it is necessary to adapt the system's operations to variations in climatic conditions for sustained effectiveness. To address the identified problem. It is proposed the use of an intelligent power management control (IPMC) system employing fuzzy logic control (FLC). The IPMC is designed to optimize the performance of energy sources and backup systems. It aims to predict and adjust the system's operating processes based on variations in climatic conditions, providing a dynamic and adaptive control strategy. The integration of FLC is specifically emphasized for its effectiveness in balancing multiple power sources and ensuring a steady and secure operation of the system. The proposed IPMC with FLC offers several advantages over existing strategies. Firstly, it showcases enhanced power availability, particularly under challenging conditions such as heavy loads or low renewable energy output. Secondly, the system protects and extends the lifespan of the power sources, contributing to long-term sustainability. The dynamic adaptation to climatic variations adds a layer of resilience to the system, making it well-suited for diverse geographical and climatic conditions. The use of realistic data and simulations in MATLAB/Simulink, along with real-time findings from the RT-LAB simulator, indicates the reliability and practical applicability of the proposed IPMC strategy. Efficient load supply and preserved batteries further underscore the benefits of the fuzzy logic-based control strategy in achieving a well-balanced and secure system operation.

## Introduction

The integration of photovoltaic (PV) solar and wind energy, along with diesel generators in off-grid or grid-connected systems, presents numerous advantages. Despite these benefits, there exists a research gap in addressing specific challenges related to intelligent power management control (IPMC) for hybrid renewable energy systems (HRES)^1^. The intermittency of solar and wind sources, the necessity for effective energy storage, and the reliance on backup sources during periods of low renewable energy output pose challenges that warrant focused investigation^2^. The motivation behind this study is to contribute to filling this research gap by proposing an innovative IPMC solution employing fuzzy logic control (FLC).

Maximum Power Point Tracking (MPPT) methods are also important for improving the efficiency of solar and wind energy systems^3^. MPPT techniques ensure that these systems run at maximum power output by constantly modifying the operating point to reflect changing environmental conditions. There is various maximum power point tracking (MPPT) strategies with different algorithms depending on the specific application. In PV systems, there exist diverse classical and advanced MPPT methods. Classical MPPT approaches are further classified as direct or indirect. Perturb and observe algorithm (P&O) method is the most commonly applied^4,5^.

Some popular advanced MPPT methods include Particle Swarm Optimization (PSO), Fuzzy Logic Control (FLC), Artificial Neural Networks (ANN), Genetic Algorithm (GA), and others. Hybrid MPPT, which is the combination of two MPPT methods, has been gaining in popularity becoming popular in recent years. For sophisticated MPPT methods, there are several approaches. The most used are the Particle Swarm Optimization (PSO)^6^, the Modified PSO (MPSO)^7,8^, the ant colony optimization (ACO)^9^, the Fuzzy logic Control (FLC)^8,10–13^, the Adaptive Fuzzy logic Control (AFLC)^8,14,15^, the Artificial Neural Networks (ANN)^16^, the Genetic algorithm (GA)^17^, the Quadratic Maximization algorithm (QM)^18^, the Cuckoo search approach (CSA)^19^, the Jaya algorithm (JA)^20^, the Grey wolf optimization (GWO) and MGO^21^, the Cat swarm optimization (CSO)^22^, etc. Hybrid MPPT (HMPPT) techniques have been used extensively during these last years. It can be a combination of two classical strategies (CMPPT-CMPPT) or a classical with an advanced one (CMPPT-AMPPT)^6,9^, or with two advanced methods (AMPPT-AMPPT)^23^. Different controllers are also used in wind turbines to track maximum power^24–40^, including classical strategies such as Perturb and Observe (P&O)^24^, Hill Climbing Search (HCS)^25^, Tip Speed Ratio (TSR)^26^, Optimal Torque Control (OTC)^27^, Power Signal Feedback (PSF)^28^ etc. Advanced methods include Fuzzy Logic Controller (FLC), Adaptive Fuzzy Logic Control (AFLC)^29^, Genetic Algorithm (GA))^30^, Adaptive Neuro-Fuzzy Inference System (ANFIS)^31^, Artificial Neural Network (ANN)^32^, Particle Swarm Optimization (PSO)^33^, Radial Basis Function Network (RBFM)^34^, Sliding Mode Control (SMC)^35^, Gradient Method (GM)^36^, Ant Colony Optimization (ACO). Hybrid MPPT methods can be combinations of two classical methods (HCS-OTC…) or a classical method and an advanced method (HCS-FLC OTC-FLC^37^, or two advanced methods^39–43^. In summary, the methods and techniques discussed earlier serve the purpose of enhancing the effectiveness of renewable energy systems. The selection of a suitable Maximum Power Point Tracking (MPPT) method depends on the unique features of the renewable energy system and the desired performance criteria.

The power management control (PMC) in a system is important in controlling the flow of energy from different sources and ensuring a stable output voltage and frequency^38,41–47^. First, Hybrid power generation systems typically combine multiple sources of energy, such as solar panels, wind turbines, fossil fuel generators, and energy storage systems. Each of these sources can have different characteristics and output profiles. In addition to that, PMC plays a pivotal role in seamlessly integrating and coordinating the energy flow from these diverse sources^42^. PMC ensures that the power generated from different sources is synchronized and controlled to meet the required output specifications^43^. Power demand in real-world scenarios is often variable and dynamic. In addition to adapting to changes in load demand and ensures that the energy sources respond effectively to maintain system stability. PMC can prioritize energy sources based on their availability and capacity to meet the demand^1,8,37^. The aforementioned strategy can make intelligent decisions about which energy sources to utilize at any given time. It considers factors like the availability of renewable energy sources (e.g., solar and wind), the cost of fossil fuel generation, and the state of charge of energy storage systems^44^. Enhancing the reliability and resilience of hybrid power systems, power management strategies can quickly adapt to changes, such as sudden cloud cover or wind fluctuations, by shifting between energy sources or adjusting their output to ensure a constant power supply^47^. A primary objective of power management techniques is the efficient utilization of available energy sources. By avoiding energy wastage and ensuring that each source operates at its optimal point, PMC enhances the overall system efficiency. The PMC can use various control strategies such as MPPT algorithms. The FLC can be used as a power management strategy in a multi-source energy system that combines photovoltaic, wind turbine, diesel generator, and storage battery. It is capable of successfully coordinating and managing energy distribution amongst these sources to increase overall system performance and efficiency. The FLC optimizes system performance by determining the most cost-effective power source to use depending on a variety of criteria such as the solar and wind power availability, the diesel fuel cost and the battery state of charge. The adaptability and decision-making capabilities offered by Fuzzy logic controllers make them valuable tool for optimizing the operation of complex hybrid power generation systems.

The need for a backup energy source in hybrid renewable energy systems (HRES) is crucial because solar irradiance, wind speed is unreliable and subject to natural variations. These variations can lead to periods of low or no energy production from these sources, and a backup energy source ensures continuous power supply even during such unpredictable conditions^48,49^. This backup source could be conventional fossil fuels or other reliable energy sources like natural gas, diesel generators or biomass that can be easily dispatched when needed, ensuring the stability and reliability of the HRES. These backup energy sources serve as a safety net, ensuring that essential services and power needs can be met when the primary renewable energy sources are not producing enough energy. However, efforts are continually being made to reduce the reliance on non-renewable backup sources by improving energy storage technologies and grid management to enhance the reliability of HRES systems while minimizing the use of backup fossil fuels^50^. Numerous studies have been conducted and published in the literature focusing on managing the energy use of electricity consumers and hybrid renewable energy sources (HRES)^50,51^. Research in this area is crucial for advancing the development and deployment of more sustainable and efficient energy systems. These studies typically aim to address various aspects of energy management, such as optimizing energy consumption, integrating renewable energy sources effectively, and improving overall energy sustainability. Some common areas of research in this field may include Demand-Side Management (DSM), Grid Integration, Energy Storage, Energy Management Systems (EMS), Economic and Environmental Analysis, Technological Advancements, (Table 1).

**Table 1:** Some common areas of research in this field of HRES.

Common areas	Operation	References
Demand-side management	To lower peak demand and energy prices, researchers are investigating how consumers can better regulate their electricity usage through strategies such as load shifting, load shedding, and energy-efficient technology	^51–54^
Grid integration	Considering grid stability, energy storage, and smart grid technologies, grid integration considers how HRES can be easily integrated into the existing electrical grid	^54–57^
Energy storage	Excess energy generated by HRES is being stored for later use using various energy storage options such as batteries, pumped hydro storage, or thermal energy storage	^1,37,43,57–60^
Energy management systems	Developing and upgrading EMS to enable effective control and monitoring of HRES energy generation, storage, and consumption	^1,8,9,36,37^
Economic and environmental analysis	Evaluating the economic and environmental viability of HRES systems and energy management strategies	^61–63^
Technological advancements	Investigating advances in renewable energy technology such as advanced solar panels, wind turbines, and other potential clean energy sources	^54,64–67^

The proposed approach is crucial because it addresses the identified research gap in HRES's intelligent power management control. It offers a dynamic and adaptive solution, optimizing energy distribution and balancing multiple sources effectively. The emphasis on reducing reliance on non-renewable backup sources aligns with sustainability goals, making the proposed IPMC a valuable contribution to the field. The comprehensive evaluation methodology ensures the practical applicability and reliability of the proposed solution in real-world scenarios. Overall, the need for the proposed approach stems from its potential to enhance the resilience, stability, and efficiency of hybrid renewable energy systems.

The hybrid power system discussed in this work comprises PV panels, a wind turbine, with a diesel generator and battery storage. This mix of energy sources allows for a more robust and versatile power generation system. The employment of a power flow or supervisory approach facilitates the management of the various power sources. This technique has been mentioned in past investigations^1,8,9,43,47^. The method is described as simple, quick, easy to implement, and does not involve heavy computations. The principal purpose of the proposed IPMC is to meet the load power needs. A secondary purpose is to keep the battery charged at a level that prevents blackouts and extends the overall lifespan of the battery. This dual-goal approach emphasizes the importance of both supplying immediate power needs and ensuring long-term stability and reliability.

Challenges arise from the variability of solar irradiation and wind availability, impacting the reliable and consistent delivery of energy. Current solutions involve backup mechanisms and energy storage, often relying on conventional fossil fuels. Efforts to minimize this dependence and enhance the reliability of HRES systems are ongoing, emphasizing the need for advanced control strategies. In this context, the proposed IPMC using FLC aims to optimize the performance of energy sources, extend their lifespan, and ensure continuous power supply. Unlike existing strategies, the IPMC considers variations in climatic conditions and efficiently balances multiple power sources. The contribution of this study lies in the development of a dynamic and adaptive IPMC solution tailored to the challenges specific to HRES. The study employs MATLAB/Simulink simulations and real-time findings from the RT-LAB simulator, providing a comprehensive evaluation. The proposed IPMC not only addresses current challenges in HRES but also contributes to the advancement of intelligent power management strategies for sustainable and efficient energy systems.

## Studied hybrid system

**Figure 1:**
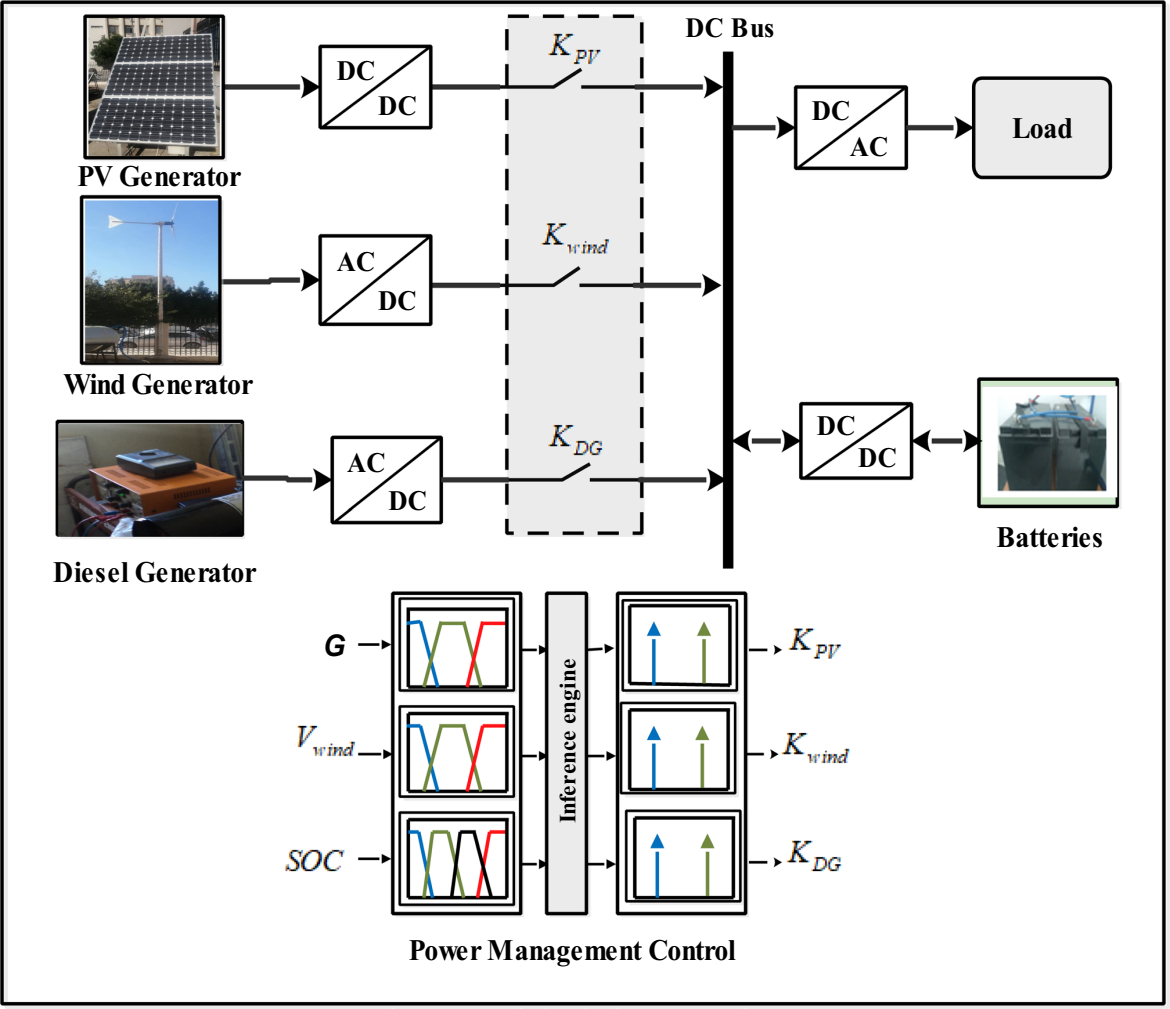
Proposed hybrid system with power management control.

The hybrid system integrates solar and wind sources, a diesel generator and batteries for storage (Fig. 1). Hybridization of wind and solar energy aims to leverage the complementary nature of these sources, considering their intermittent nature. A diesel backup generator is included in the system to provide additional power during low energy production or high demand, ensuring continuous power availability. Also, batteries play a crucial role in storing excess energy during times of high renewable energy production and releasing it when energy demand exceeds the current production. Diesel backup generators and batteries help to ensure a steady and reliable power supply, especially during times when renewable energy is scarce. The combination of wind and solar energy sources, coupled with backup capabilities from the diesel generator and energy storage, provides a more robust and resilient power generation system.

### Photovoltaic model

Mathematical models are quite important in understanding and predicting the behavior of photovoltaic (PV) generators. The model to be used is determined by the amount of precision required, the complexity of the simulation, and the data available for parameterization. Each model has its strengths and limitations^68,69^. Table 2 gives the advantages and the drawbacks of the different types of mathematical models commonly used in PV generator modeling, each with its specific focus and application.

**Table 2:** Advantages and drawbacks of different types of mathematical models^8^.

Model type	Advantages	Drawbacks
One-diode model	Simplicity and ease of implementation Suitable for many applications Computationally efficient	Does not capture all aspects of complex behavior (e.g., partial shading effects)
Double-diode model	Improved accuracy better representation under challenging conditions, such as partial shading	Increased complexity and computational requirements Requires more parameters for characterization
Empirical models	Flexibility to fit a wide range of experimental data Simple implementation	Lack of physical insight Limited extrapolation capability beyond the range of experimental data
Analytical models	Detailed physical insights into PV cell behavior High potential for accuracy when parameters are well-known	High computational complexity May require detailed knowledge of material properties and conditions
Equivalent circuit models	Simplicity and efficiency in system-level simulations Suitable for understanding overall system behavior	Simplified representation may not capture all aspects of PV cell behavior Accuracy depends on the chosen equivalent circuit and parameters
Temperature and irradiance models	Specific focus on environmental impact, providing insights into temperature and irradiance effects Simplicity in integration with other models	Limited in capturing other complex behaviors of the PV cell Accuracy highly dependent on the quality of environmental data and model parameters
Two-diode models	Improved accuracy compared to the single-diode model Enhanced representation under challenging conditions	Higher computational requirements May require additional parameters, which could be challenging to determine experimentally

**Figure 2:**
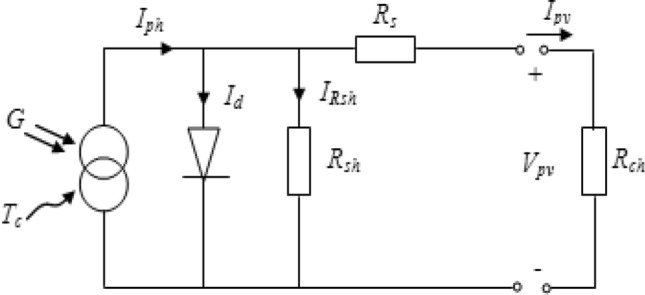
PV one diode model.

The one-diode model is commonly used in PV system modeling for several practical reasons as simplicity and accuracy, so it is used in our work. In this case, the electrical current is (Fig. 2)^1,8^:

1 $${{\text{I}}}_{{\text{pv}}}= {{\text{I}}}_{{\text{ph}}}-{{\text{I}}}_{{\text{d}}}-{{\text{I}}}_{{\text{Rsh}}}$$

with: I_ph_ the photo-current, I_d_ the diode-current and I_Rsh_ the shunt resistance R_sh_.

**Figure 3:**
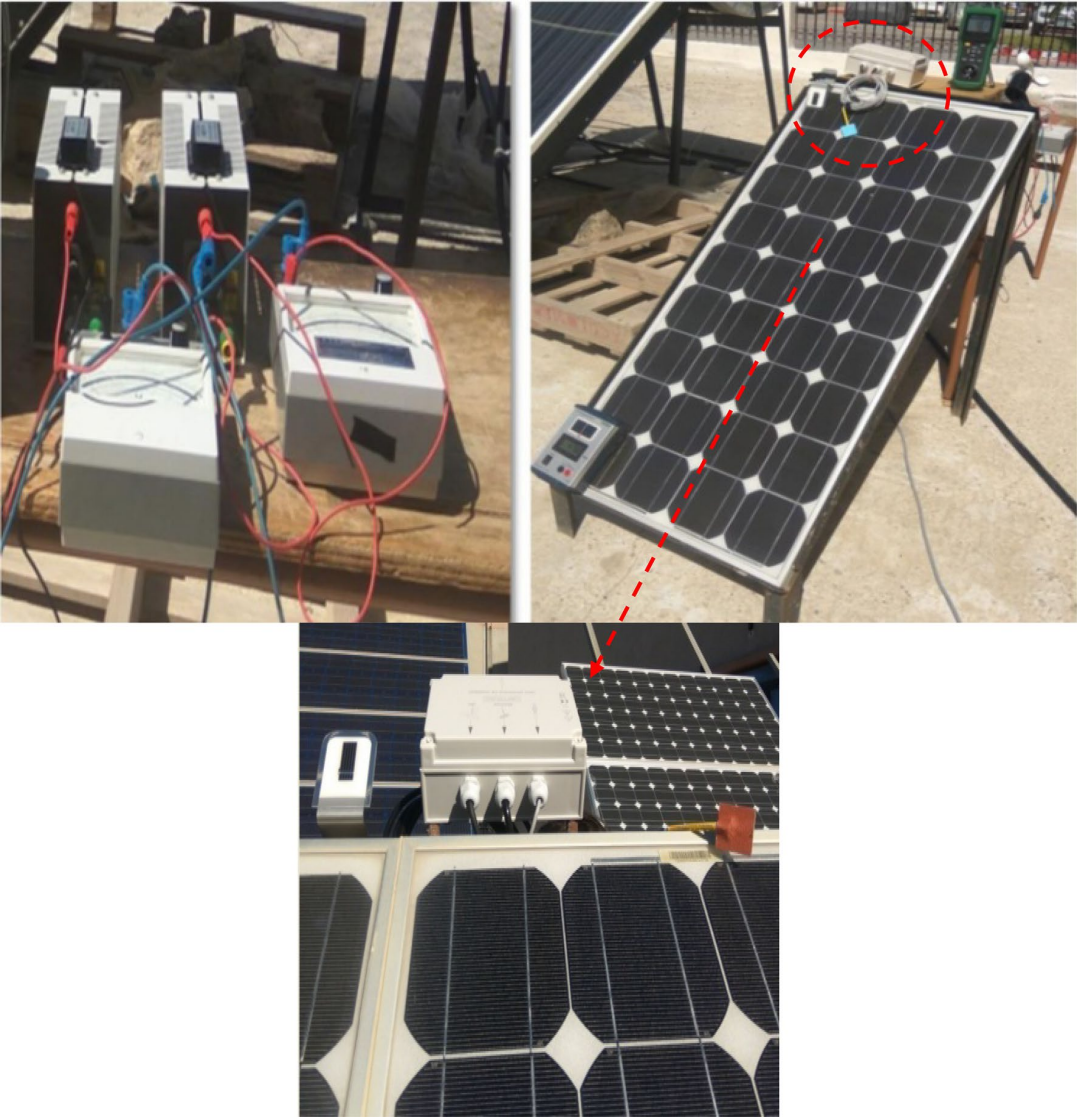
Determination of the electrical characteristics.

**Figure 4:**
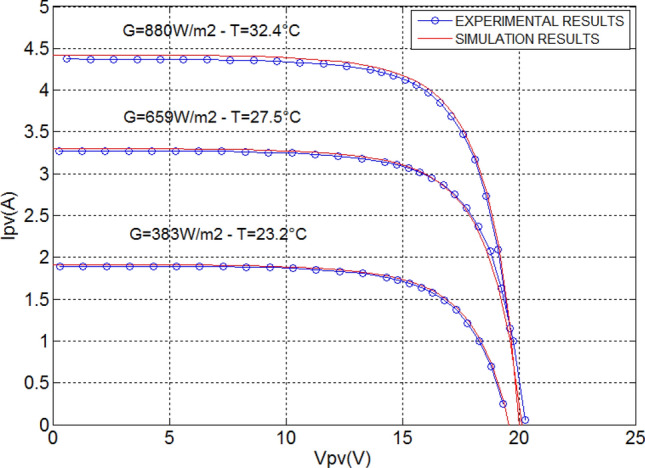
I_pv_(V_pv_) curves (simulation and experimental).

**Figure 5:**
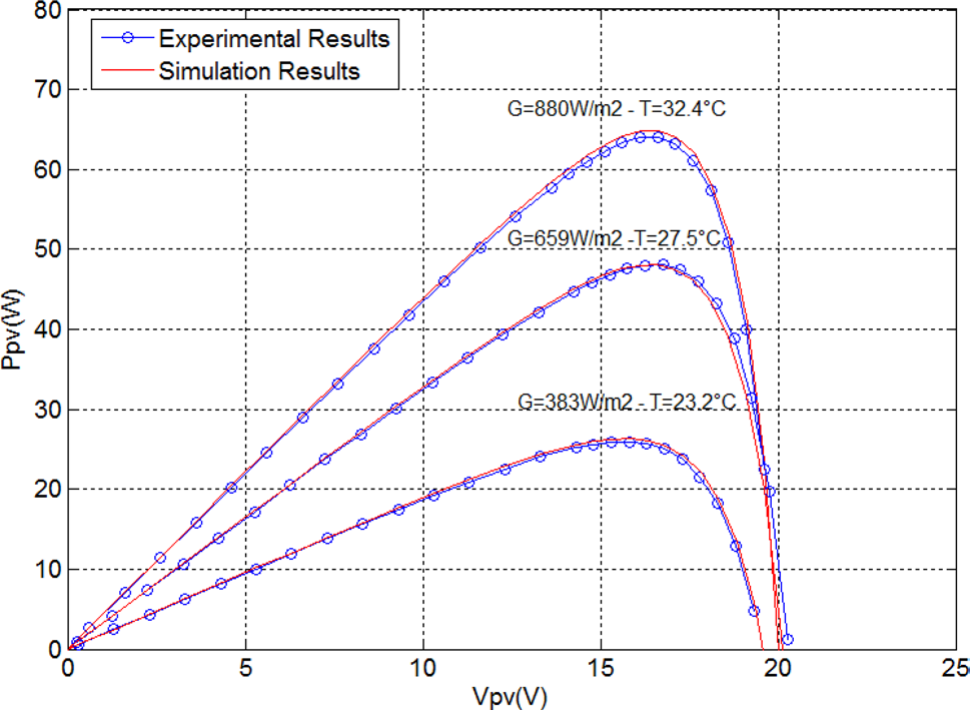
P_pv_(V_pv_) curves (simulation and experimental).

Based on experimental tests (Fig. 3), the parameters of a PV panel have been determined (Figs. 4 and 5) utilizing the electrical properties of PV (80Wp)^8^. Measurement sensors was used to measure the sun radiation, and temperature, to transfer the different signals to a data processing interface and then to a PC where they will be displayed using ACQUIsol software in real-time.

The established experimental bench is composed by an 80Wp panel (Table 3.), a voltmeter and an amperemeter with a variable load. The ambient temperature and solar irradiance are measured by using measurements devices. Extensive numerical simulations were carried out under MATLAB/Simulink environment. Runge Kutta of 4th order is used as a solver with a step of 1e−5.

**Table 3:** Parameters of the photovoltaic panel 80 Wp.

Parameters	Values
Photovoltaic power P_pv_	80 Wp
Maximum current at PPM I_mpp_	4.65 A
Maximum voltage at PPM V_mpp_	17.5 V
Short circuit current I_sc_	4.95 A
Open circuit voltage V_oc_	21.9 V
Temperature coefficient of short-current α_sc_	3 mA/°C
Voltage temperature coefficient of short-current Β_oc_	− 150 mV/°C

### Wind turbine modeling

**Figure 6:**
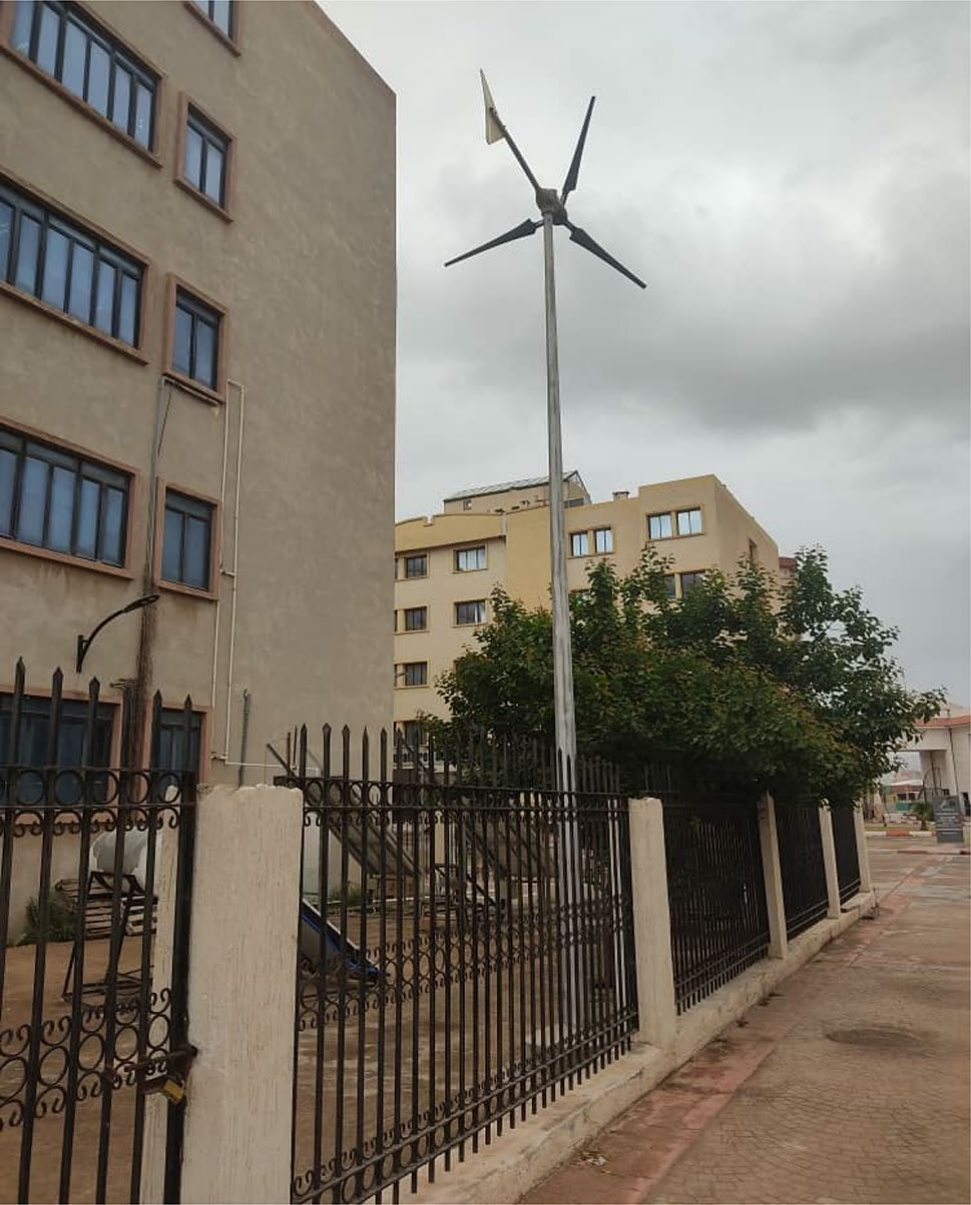
Used wind turbine of 900 W.

The system shown in Fig. 6. includes a wind turbine, suggesting the use of wind energy to drive a permanent magnet synchronous generator (PMSG). The different equations are^10,27,28^:

2 $${P}_{wind}=\frac{1}{2}{C}_{p}\left(\lambda \right) \cdot {\rho }_{air} \cdot \pi \cdot {R}^{3} \cdot {V}_{wind}^{3}$$

3 $${T}_{wind}={T}_{mec}=\frac{1}{2}\frac{{C}_{p}\left(\lambda \right) \cdot {\rho }_{air} \cdot \pi \cdot {R}^{2} \cdot {V}_{wind}^{2}}{\uplambda }$$

4 $${C}_{p}=\frac{2 {P}_{wind}\left(\lambda \right)}{\lambda \cdot \pi \cdot {R}^{2} \cdot {V}_{wind}^{3}}$$

where *C*_*p*_ is the power coefficient, V_wind_ the wind speed, *λ* the tip speed ratio, R the radius of the rotor radius, and *ρ* the density of the air.

The voltage equations are given as^37,38^:

5 $$\left\{\begin{array}{l}{{\text{V}}}_{{\text{ds}}}= {{\text{R}}}_{{\text{s}}} \cdot {{\text{I}}}_{{\text{ds}}}+{{\text{L}}}_{{\text{ds}}}\frac{{{\text{dI}}}_{{\text{ds}}}}{{\text{dt}}}-{\text{P}} \cdot \upomega \cdot {{\text{L}}}_{{\text{qs}}} \cdot {{\text{I}}}_{{\text{qs}}}\\ {{\text{V}}}_{{\text{qs}}}= {{\text{R}}}_{{\text{s}}} \cdot {{\text{I}}}_{{\text{qs}}}+{{\text{L}}}_{{\text{qs}}}\frac{{{\text{dI}}}_{{\text{qs}}}}{{\text{dt}}}+{\text{P}} \cdot \upomega \cdot {{\text{L}}}_{{\text{ds}}} \cdot {{\text{I}}}_{{\text{ds}}}+{\text{P}} \cdot \upomega \cdot {\upphi }_{{\text{f}}}\end{array}\right.$$

where: V_ds_ and V_qs_ are the stator voltages with the direct and quadrate axis, R_s_ the stator winding resistance, I_ds_ and I_qs_ the stator currents with the direct and quadrate axis, L_ds_ and L_qs_ are the inductances with the direct and quadrate axis, P the number of pole pairs, ω the angular velocity, and Φ_f_ the magnetic flux produced by the permanent magnet^70,71^.

The electromagnetic torque is written as^37,38^:

6 $${T}_{em}=\frac{3}{2}{\text{P}}\lceil\left({{\text{L}}}_{{\text{ds}}}-{{\text{L}}}_{{\text{qs}}}\right){{\text{I}}}_{{\text{ds}}} \cdot {{\text{I}}}_{{\text{qs}}}+{\phi }_{f} \cdot {{\text{I}}}_{{\text{qs}}}\rceil$$

### Battery storage modeling

**Figure 7:**
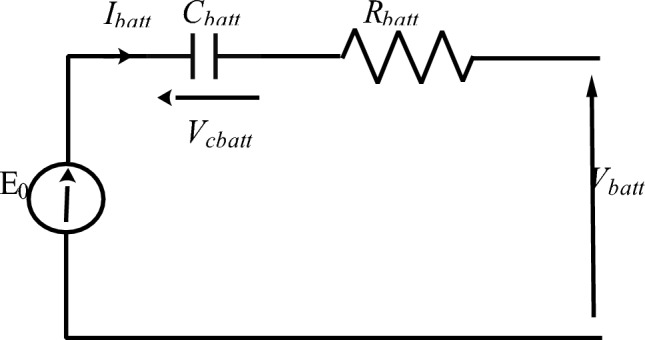
Equivalent battery electrical circuit.

The models can be used to simulate different scenarios and determine the most efficient and cost-effective ways to use the battery storage in conjunction with the other power sources^72,73^. Figure 7 depicts the model utilized in this investigation^1,8,9^:

7 $${{\text{V}}}_{{\text{batt}}}={{\text{E}}}_{{\text{batt}}}\pm {{\text{R}}}_{{\text{batt}}} \cdot {{\text{I}}}_{{\text{batt}}}$$

where V_batt_ is the battery voltage, E_batt_ open circuit voltage, R_batt_ internal battery resistance and I_batt_ battery current.

**Figure 8:**
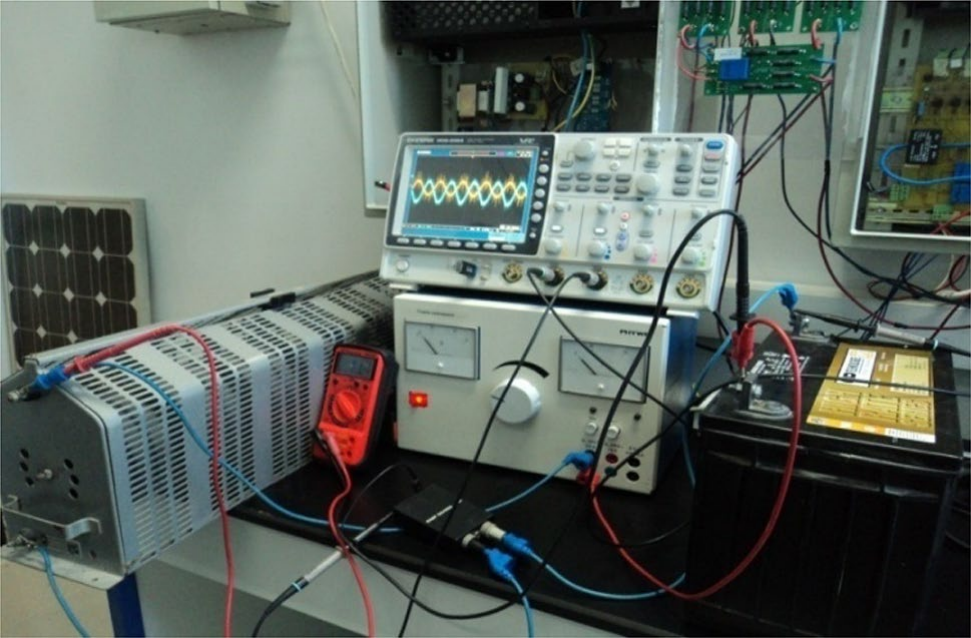
Battery identification test.

An identification of the battery used of 12 V-100 Ah was carried out in the laboratory (Fig. 8). The battery is considered as an impedance Z_batt_ with a resistance R_batt_ and a reactance X_batt_.

8 $${{\text{Z}}}_{{\text{batt}}}=\frac{{{\text{U}}}_{{\text{batt}}}}{{{\text{I}}}_{{\text{batt}}}}$$

9 $$\left\{\begin{array}{l}{{\text{R}}}_{{\text{batt}}}={{\text{Z}}}_{{\text{batt}}} \cdot \mathrm{cos\rho }\\ {{\text{X}}}_{{\text{batt}}}={{\text{Z}}}_{{\text{batt}}} \cdot \mathrm{sin\rho }\\ {{\text{C}}}_{{\text{batt}}}=\frac{1}{\left({{\text{X}}}_{{\text{batt}}} \cdot 2 \cdot \uppi \cdot {\text{f}}\right)}\end{array}\right.$$

with ƒ the frequency (Hz).

The acquired results are R_batt_ = 0.756 Ω and X_batt_ = 0.072 Ω.

### Diesel generator (DG) modeling

**Figure 9:**
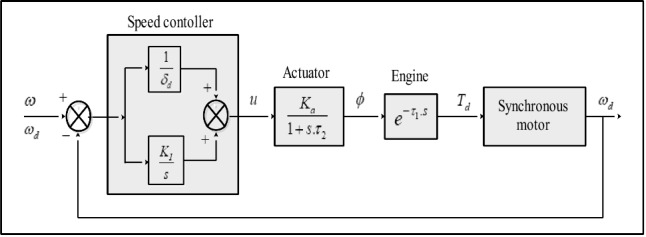
Dynamic model of diesel generator.

The complete diesel generator dynamic model involves modeling both the diesel engine with its speed control loop and the synchronous generator with its voltage control system (Fig. 9).

The rotational speed error is the input of the speed controller, and the actuator control signal is its output. The droop *δ*_*d*_ and the integrator factor K_I_ are the parameters of the speed controller^74,75^. The goal of the integrator is to eliminate the static speed error. A first-order model with the gain K_a_ and a time constant *τ*_*2*_ is used to approximate the operational dynamics of the actuator. The fuel temperature affects this time factor. Although K_a_ and *τ*_*2*_ are both variable, their variation is negligible for short time periods. The equation of the synchronous motor mechanical is:

10 $${{T}_{d}-T}_{em}=J \cdot \frac{d{\Omega }_{m}}{dt}$$

With J the motor inertia, ${\Omega }_{m}$ the rotationnal speed, T_d_ the diesel mechanical torque and T_em_ the electromagnetic torque.

## Sizing of the studied system

To obtain the appropriate size of each power source, such as the photovoltaic panels and wind turbine, the energy generation during each month of PV and wind generator and the load demand are calculated^8^. The PV and wind turbine generator areas are calculated from the ratios of the monthly energies:

11 $${{\text{A}}}_{{\text{pv}}}={\text{max}}\left(\frac{{{\text{E}}}_{{\text{Load}},{\text{m}}}}{{{\text{E}}}_{{\text{pv}},{\text{m}}}}\right)$$

12 $${{\text{A}}}_{{\text{wind}}}={\text{max}}\left(\frac{{{\text{E}}}_{{\text{Load}},{\text{m}}}}{{{\text{E}}}_{{\text{wind}},{\text{m}}}}\right)$$

Then:

13 $${{\text{E}}}_{{\text{pv}}}={\upeta }_{{\text{pv}}}\cdot {{\text{A}}}_{{\text{pv}}} \cdot {{\text{E}}}_{{\text{ir}}}$$

with:

14 $${\upeta }_{{\text{pv}}}={\upeta }_{{\text{pv}}-{\text{STC}}} \cdot \left[1-{\upbeta }_{{\text{oc}}} \cdot ({{\text{T}}}_{{\text{j}}}-{{\text{T}}}_{{\text{j}}-{\text{STC}}})\right]$$

15 $${{\text{E}}}_{{\text{wind}}}={{\text{P}}}_{{\text{wind}}} \cdot \Delta \mathrm{t }=\left(1/2\right) \cdot \uprho \cdot {\text{S}} \cdot {{\text{V}}}_{{\text{wind}}}^{3} \cdot {{\text{C}}}_{{\text{p}}} \cdot \Delta {\text{t}}$$

16 $${{\text{E}}}_{{\text{Load}}}={{\text{A}}}_{{\text{pv}}} \cdot {{\text{E}}}_{{\text{pv}}}+{{\text{A}}}_{{\text{wind}}} \cdot {{\text{E}}}_{{\text{wind}}}$$

The monthly energies produced are:

17 $$\left\{\begin{array}{l}{{\text{E}}}_{{\text{pv}},{\text{ave}}}= \frac{\left({\sum }_{{\text{m}}=1}^{12}{{\text{E}}}_{pv}\right)}{12}\\ {{\text{E}}}_{{\text{wind}},{\text{ave}}}=\frac{\left({\sum }_{{\text{m}}=1}^{12}{{\text{E}}}_{wind}\right)}{12}\\ {{\text{E}}}_{{\text{Load}},{\text{ave}}}=\frac{\left({\sum }_{{\text{m}}=1}^{12}{{\text{E}}}_{Load}\right)}{12}\end{array}\right.$$

The PV and wind generators areas will be finally:

18 $$\left\{\begin{array}{l}{{\text{A}}}_{{\text{pv}},{\text{ave}}}={{\text{k}}}_{{\text{perc}}}\left({{\text{E}}}_{{\text{Load}}}/{{\text{E}}}_{{\text{pv}}}\right)\\ {{\text{A}}}_{{\text{wind}},{\text{ave}}}=\left(1-{{\text{k}}}_{{\text{perc}}}\right)\left({{\text{E}}}_{{\text{Load}}}/{{\text{E}}}_{{\text{wind}}}\right)\end{array}\right.$$

where k_perc_ and (1 − k_perc_) are respectively the fraction of the PV source and the fraction of the wind source^76^.

19 $$\left\{\begin{array}{l}{{\text{N}}}_{{\text{pv}}}=\frac{{{\text{A}}}_{{\text{pv}},{\text{ave}}}}{{{\text{A}}}_{{\text{pv}},{\text{unit}}}}\\ {{\text{N}}}_{{\text{wind}}}=\frac{{{\text{A}}}_{{\text{wind}},{\text{ave}}}}{{{\text{A}}}_{{\text{wind}},{\text{unit}}}}\end{array}\right.$$

Finally, the calculated average load is determined by:

20 $${\text{E}}_{{{\text{load,ave-cal}}}}={{\text{E}}}_{{\text{pv}},{\text{ave}}} \cdot {{\text{A}}}_{{\text{pv}},{\text{unit}}}+{{\text{E}}}_{{\text{wind}},{\text{ave}}} \cdot {{\text{A}}}_{{\text{wind}},{\text{unit}}}$$

The different findings are given in Tables 4 and 5.

**Table 4:** Energies calculations.

Months	E_ir_ (kWh/m^2^)	E_pv_ (kWh/m^2^)	E_wind_ (kWh/m^2^)	E_Load_ (kWh)
January	160.5370	11.5587	14.9655	121.52
February	163.8860	11.7998	13.8975	113.68
March	187.6466	13.5106	14.3165	121.52
April	185.2149	13.3355	12.7508	117.60
May	190.0646	13.6847	11.3513	121.52
June	190.4512	13.7125	12.2691	117.60
July	201.1160	14.4804	13.2769	121.52
August	203.5266	14.6539	13.8944	121.52
September	193.1726	13.9084	11.2524	117.60
October	182.3539	13.1295	12.2890	121.52
November	157.0889	11.3104	12.9470	117.60
December	151.4980	10.9079	17.8809	121.52
Monthly average	180.5464	12.9993	13.4243	**119.56**

Significant values are in bold.

**Table 5:** Panels and wind turbine number calculation.

${{\text{k}}}_{{\text{perc}}}$	A_pv_ (m^2^)	A_wind_ (m^2^)	N_pv_	N_wind_	E_load,ave-cal_ (kWh)
0	0	9.42	0	3	126.46
0.1	1.292	9.42	2	3	143.25
0.2	1.938	9.42	3	3	151.65
0.3	3.230	9.42	5	3	168.44
0.4	3.876	6.28	6	2	134.69
0.5	5.168	6.28	8	2	151.48
0.6	5.814	6.28	9	2	159.88
0.7	7.106	3.14	**10**	**1**	**134.53**
0.8	7.752	3.14	12	1	142.92
0.9	8.3980	3.14	13	1	151.32
1	9.6900	0	15	0	125.96

It can be concluded that only (10 panels and 01 wind turbine) configuration can be considered.

The serial PV calculation is:

21 $${\text{N}}_{{{\text{pv-serial}}}}=\frac{{{\text{E}}}_{{\text{Load}}}}{{{\text{E}}}_{{\text{worst}}}{{\text{K}}}_{{\text{loss}}}}$$

$${\text{N}}_{{{\text{pv-serial}}}}=\frac{4.33}{3.5.0.65}=2{\text{panels}}$$

And PV maximum voltage will be:

22 $${\text{V}}_{{{\text{pv-max}}}}=1.5{{\text{N}}}_{{\text{pv}}-{\text{serial}}}{{\text{U}}}_{{\text{oc}}}$$

$${\text{V}}_{{{\text{pv-max}}}}=1.5.\mathrm{2.21.9}=50.37{\text{V}}$$

Thus the number of strings is:

23 $${\text{N}}_{{{\text{pv-string}}}}=\frac{{{\text{U}}}_{{\text{pv}}-{\text{max}}}}{{{\text{V}}}_{{\text{DC}}}}$$

$${\text{N}}_{{{\text{pv-string}}}}=\frac{50.37}{12}=5\;{\text{panels}}$$

With ${{\text{E}}}_{{\text{worst}}}$ the worst solar energy irradiation at the studied site (3.5 kWh/m^2^ day) and ${{\text{K}}}_{{\text{loss}}}$ represent the different losses.

Finally, the outcome is 5 strings.

The battery capacity is^77,78^:

24 $${{\text{C}}}_{{\text{batt}}}=\frac{\left({{\text{d}}}_{{\text{aut}}}{{\text{E}}}_{{\text{load}},{\text{m}}}\right)}{\left({{\text{U}}}_{{\text{Batt}}}{\text{PDP}}{\upeta }_{{\text{batt}}}{{\text{N}}}_{{\text{m}}}\right)}$$

$${{\text{C}}}_{{\text{batt}}}=\frac{\left(3.134\times 53.1000\right)}{\left(12.0\times .8.0.\times 9.31\right)}=1506.83 \; {\text{Ah}}$$

With ${d}_{aut}$ the days of autonomy (days),${{\text{E}}}_{{\text{load}},{\text{m}}}$ the consumed monthly load (kWh/day), N_m_ is equal to 31 days,${{\text{U}}}_{{\text{batt}}}$ the voltage battery (V), ${\text{PDP}}$ the depth of discharge and ${\upeta }_{{\text{batt}}}$ the efficiency of the battery^79,80^.

The number of batteries can be calculated as:

25 $${{\text{N}}}_{{\text{batt}}}={\text{ENT}} \left[ {\frac{{{\text{C}}_{{{\text{batt}}}} }}{{{\text{C}}_{{{\text{batt-u}}}} }}} \right]$$

$${{\text{N}}}_{{\text{batt}}}=ENT\left[\frac{1506.83}{192}\right]=8 \;\mathrm{ batteries}$$

With ${C}_{batt{\text{-}}u}$ the chosen battery capacity.

In our study, we have chosen a DG that delivers a constant voltage of 220 V, a current of 10 A and a power of 2 kVA. Table 6 summarizes all of the quantities that will be used.

**Table 6:** Components number.

D_energy_ (kWh/day)	PV panels	Wind turbine	Batteries	DG
4.33	10 panels $2 \; {\text{serial}}$ 5 strings	1 turbine 900 W 1.05 m	8 batteries 192 Ah 12 V	1 DG 220 V 10 A 2kVA

## Proposed intelligent power management control

The management method for autonomous hybrid systems is designed to fulfill load demand and control the power flow while offering the efficient operation of all energy sources. The IPMC approach prioritizes the use of photovoltaic and wind powers to meet the load requirement and relies on the use of long-term storage to supply the load. This helps reduce the start/stop cycles of a diesel generator which can indeed lead to lower fuel consumption and improve the energy balance of the system. By operating the generator for larger periods of time at a steady state, the energy losses that occur during the start-up and the shutdown can be minimized. Additionally, the load profile of the generator can be optimized to match the electricity demand, which can further improve fuel efficiency and reduce wear and tear on the generator. This is an important part of useful energy management because it can help to reduce running costs while also lowering the environmental impact of the system. The management approach is based on a cycle in which the diesel generator is turned off until the level of charge in the battery storage reaches a minimum, then the latter is restarted and continues running until the level of charge in the battery storage reaches a maximum, and the cycle is repeated. The equation of power balance is:

26 $${{\text{P}}}_{{\text{load}}}={{\text{P}}}_{{\text{PV}}}+{{\text{P}}}_{{\text{wind}}}+{{\text{P}}}_{{\text{DG}}}\pm {{\text{P}}}_{{\text{batt}}}$$

**Figure 10:**
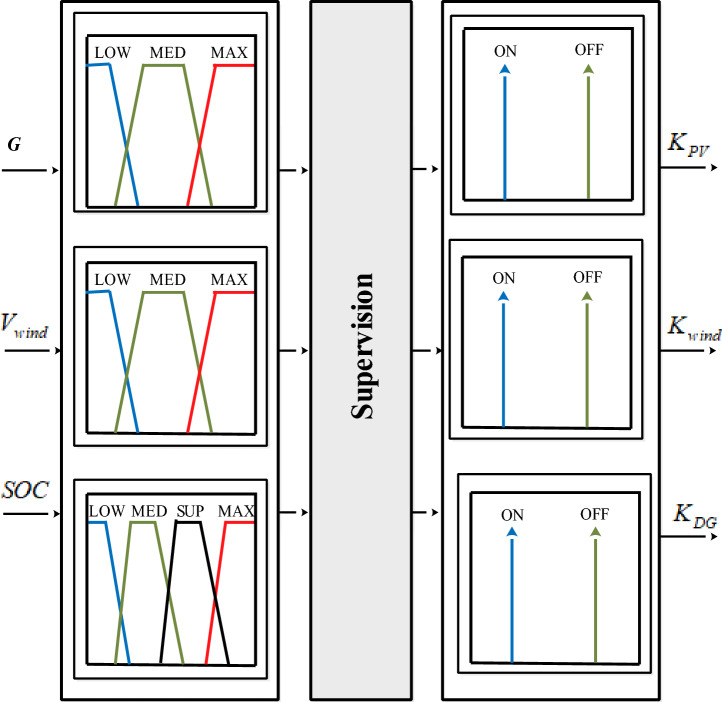
Proposed intelligent PMC of the studied system.

The use of fuzzy logic improves overall system performance and efficiency through effective coordination and management of energy distribution. It can make cost-effective decisions on power source usage, optimize battery operation, and provide a stable and reliable power supply by coordinating the power sources, the diesel generator and the battery. The primary operation of the FLC is to create three control signals from three inputs (Fig. 10). The Mamdani methodology was used to build the fuzzy inference in this work^8^.

The inputs of the fuzzy regulator are listed in Table 7.

**Table 7:** Input of the fuzzy regulator.

Input variables		Output variables	
G (W/m^2^)	Solar irradiation	K_pv_	PV control signal
V_wind_ (m/s)	Wind speed	K_wind_	Control signal for wind turbine
SOC (%)	State of charge	K_DG_	Control signal for DG

As illustrated in Table 8, it generates eight unique modes. Tables 9 and 10 indicate the relationship between each regulator input and the linguistic variables representing the fuzzy sets.

**Table 8:** The various operating modes.

Modes	Sources	P_Load_	K_pv_	K_w_	K_DG_
Mode1	PV + WTb + DG	P_load_ = P_pv_ + P_wind_ + P_DG_ − P_batt_	1	1	1
Mode2	PV + WTb	P_load_ = P_pv_ + P_wind_ − P_batt_	1	1	0
Mode3	PV	P_load_ = P_pv_ − P_batt_	1	0	0
Mode4	PV + WTb	P_load_ = P_pv_ + P_DG_ − P_batt_	1	0	1
Mode5	WTb	P_load_ = P_wind_ − P_batt_	0	1	0
Mode6	WTb + DG	P_load_ = P_wind_ + P_DG_ − P_batt_	0	1	1
Mode7	DG	P_load_ = P_DG_	0	0	1
Mode8	Batteries	P_load_ = − P_batt_ or P_load_ = 0	0	0	0

**Table 9:** Fuzzy regulator rules.

SOC	E_s_	V_wind_	K_pv_	K_wind_	K_DG_
Low	Low	Low	Off	On	Off
	Low	Med	Off	Off	Off
	Low	Max	Off	On	Off
	Med	Low	Off	On	Off
	Med	Med	Off	Off	Off
	Med	Max	Off	On	Off
	Max	Low	Off	On	Off
	Max	Med	Off	Off	Off
	Max	Max	Off	On	Off
	Low	Low	Low	Low	Low
Med and Sup	Low	Low	Low	Low	Low
	Low	Low	Low	Low	Low
	Low	Low	Low	Low	Low
	Low	Low	Low	Low	Low
	Low	Low	Low	Low	Low
	Low	Low	Low	Low	Low
	Low	Low	Low	Low	Low
	Low	Low	Low	Low	Low
Max	∀ E_s_	∀V_wind_	On	On	ON

**Table 10:** Fuzzy regulator inputs.

E_s_ (W/m^2^)	0–200	200–600	600–1000	
	Low	Med	Max	
V_wind_ (m/s)	0–3	3–12	12–20	
	Low	Med	Max	
SOC (%)	0–25	25–75	75–99	100
	Low	med	sup	max

## Simulation study

**Figure 11:**
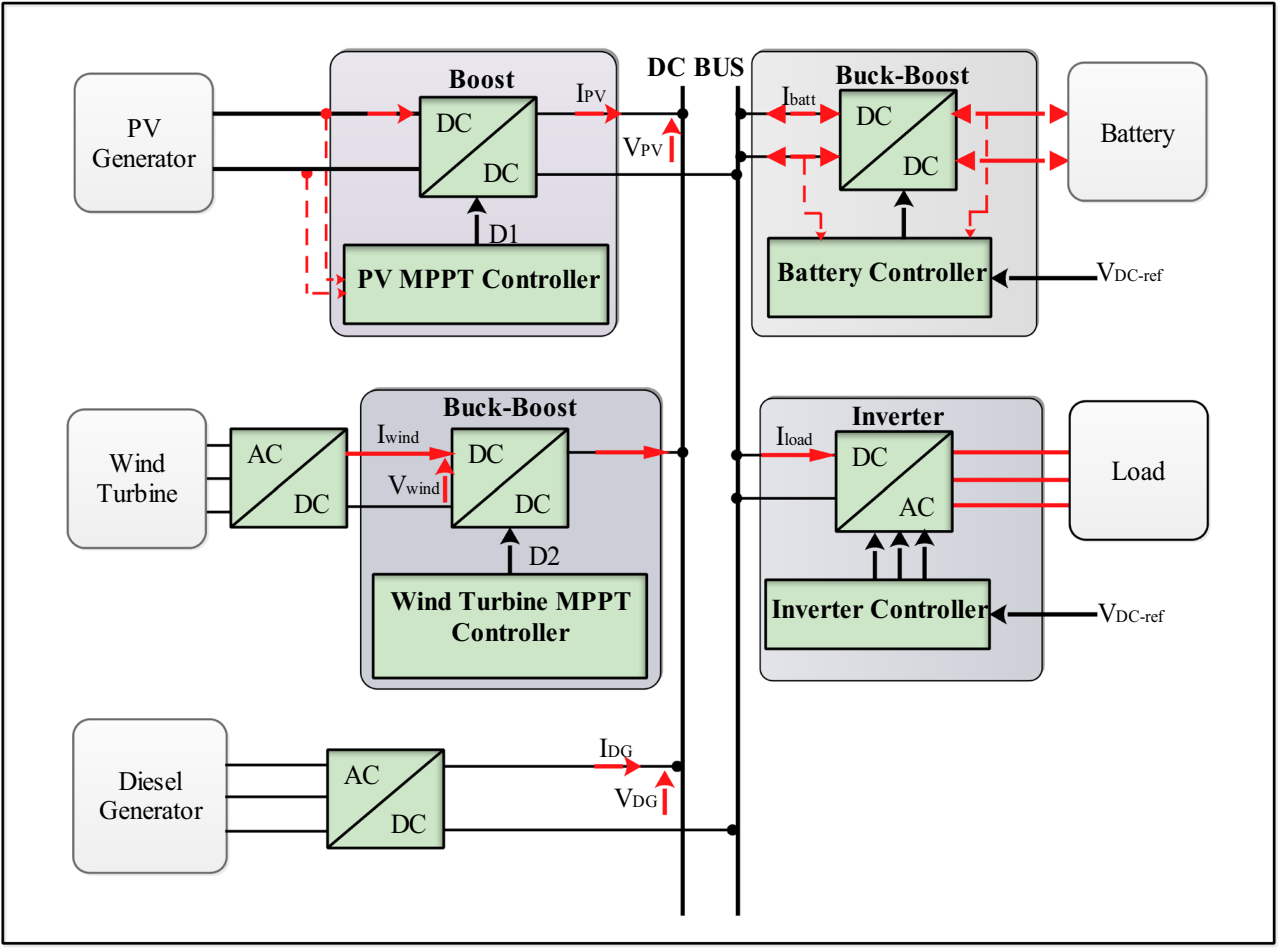
Proposed control scheme.

The controls used are designed to ensure that the voltages of PV panels and wind turbines are equal to the DC bus voltage. This helps to stabilize the system and extract the greatest amount of power, regardless of solar irradiance and wind speed variations. The control algorithms work to coordinate the power exchange between the various sources to ensure a stable and reliable power supply (Fig. 11).

**Figure 12:**
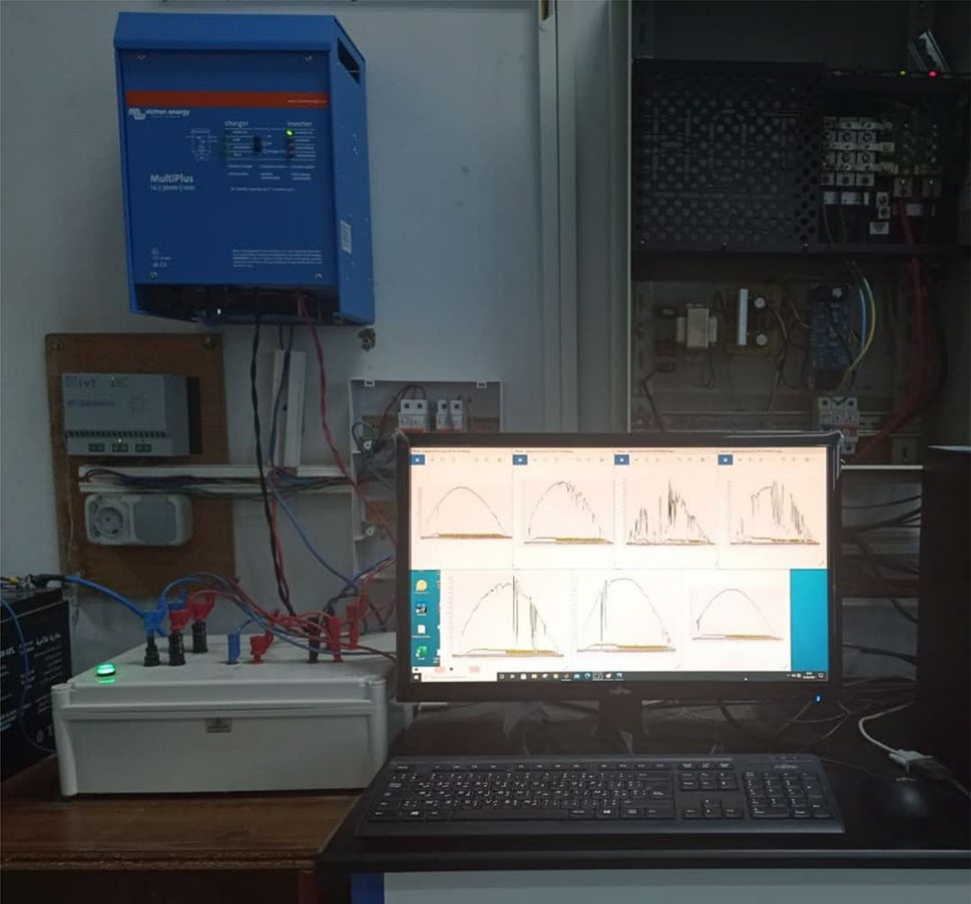
Measurement acquisition device at the laboratory.

**Figure 13:**
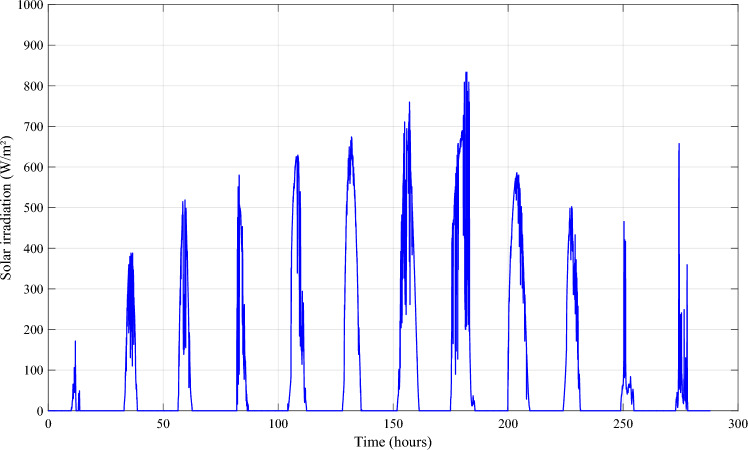
Solar irradiation profile.

**Figure 14:**
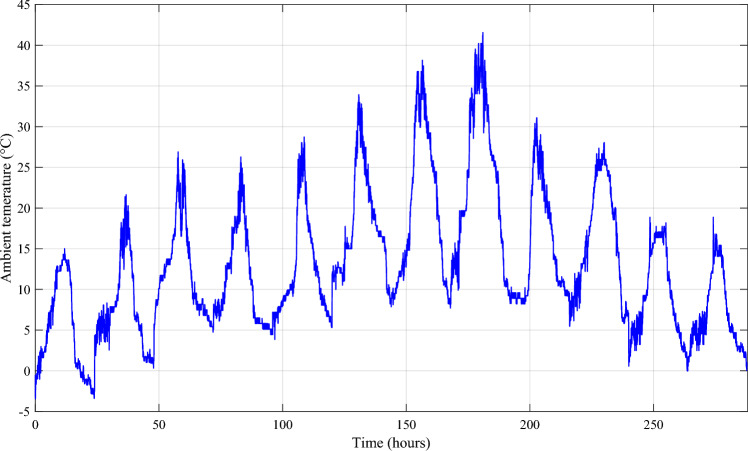
Ambient temperature profile.

**Figure 15:**
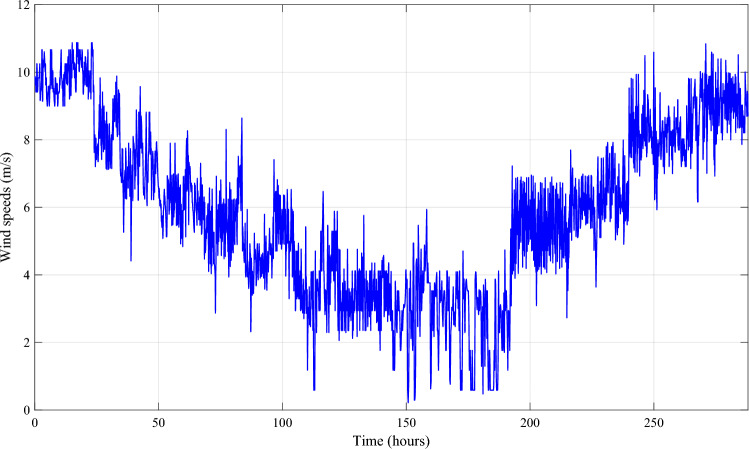
Profile of the wind speed.

Solar irradiation, ambient temperature, and wind speeds were measured using measurement acquisition equipment in the lab (Fig. 12). We have incorporated the recorded data from sun irradiation (Fig. 13), ambient temperature (Fig. 14), and wind speed (Fig. 15) in MATLAB/Simulink.

**Figure 16:**
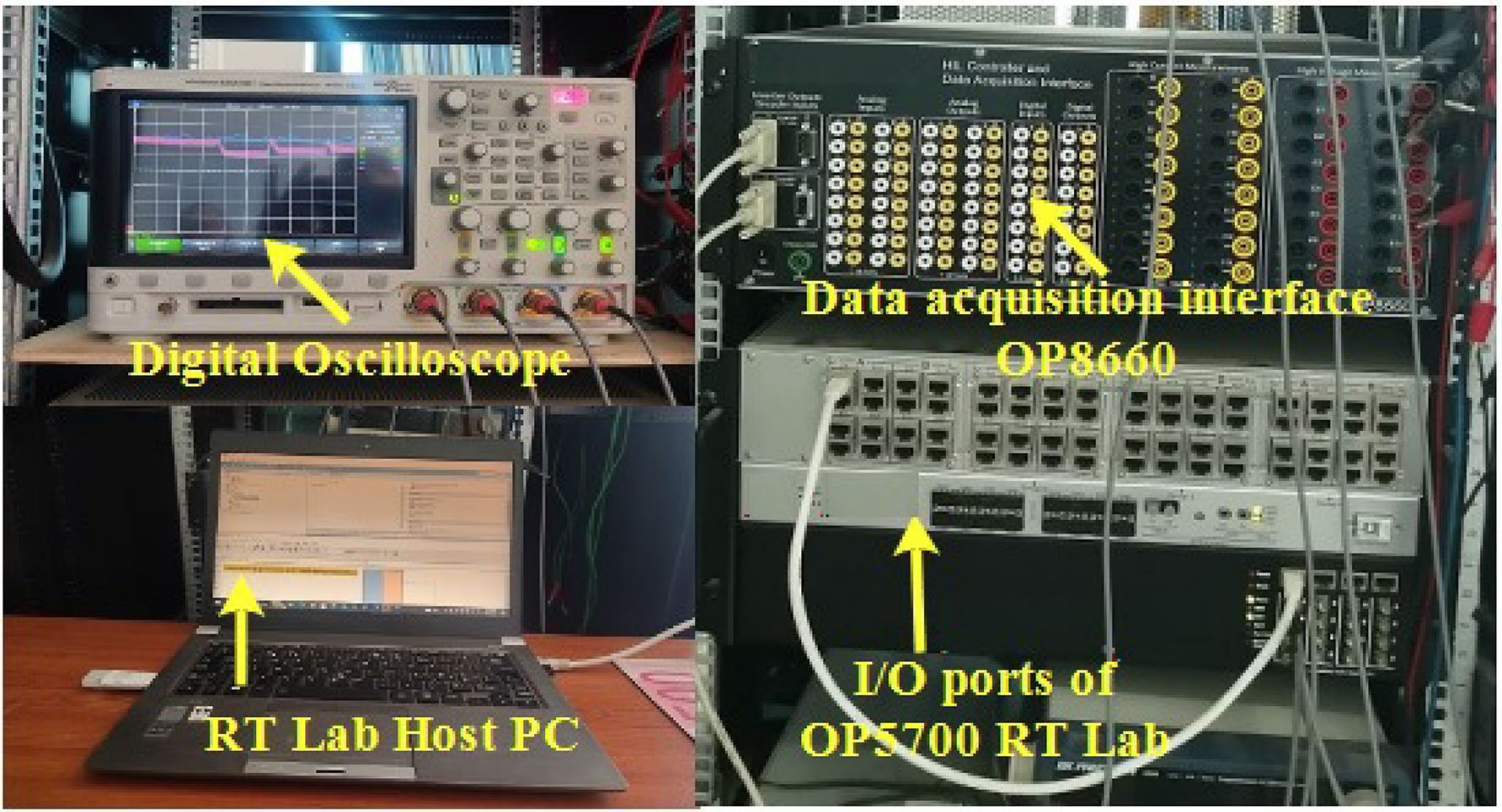
Real-time simulation bench setup.

The OPAL RT LAB simulator is used for the studied system in real-time (Fig. 16). Simulations are run in Matlab/Simulink and then in real-time.

**Figure 17:**
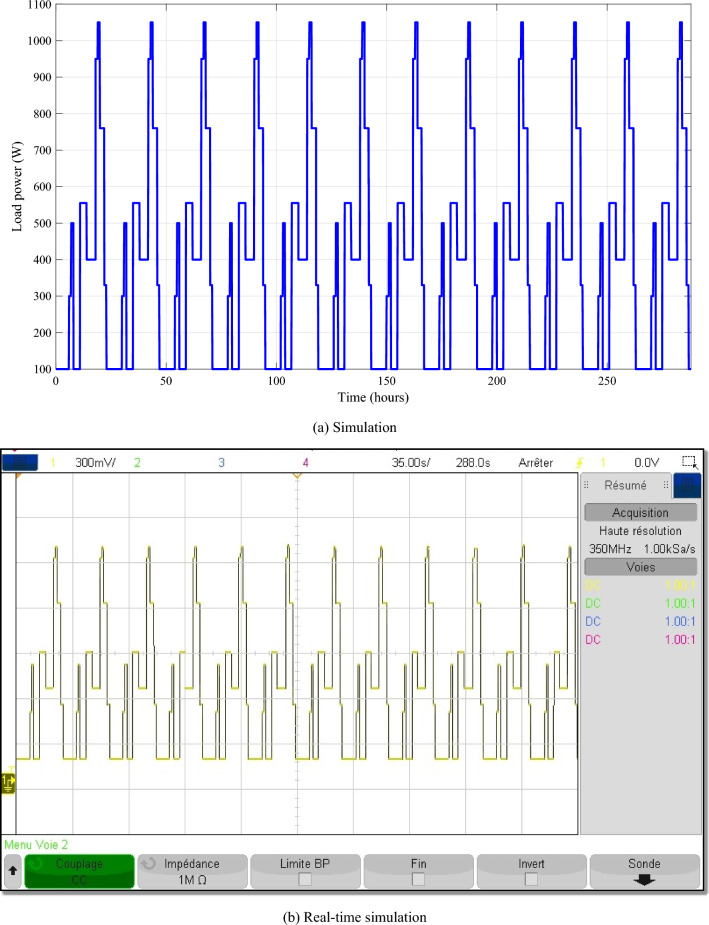
Profile of the load power.

The load power is represented as follows (Fig. 17).

**Figure 18:**
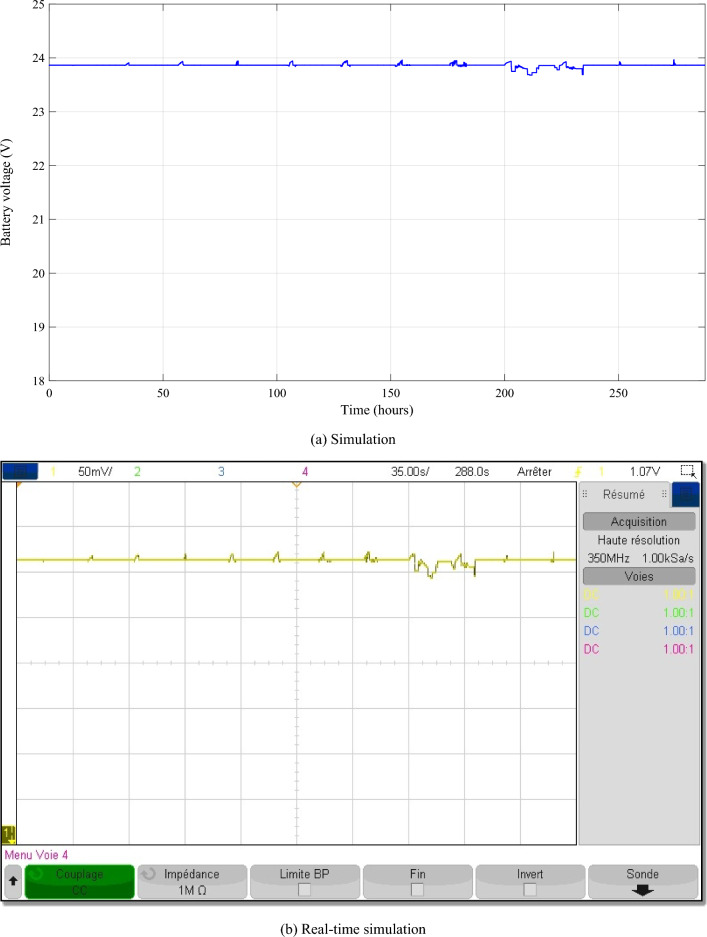
Battery voltage.

Figure 18 presents the simulated voltage profile of the battery. The battery's voltage varies in accordance with the power absorbed/injected into the DC bus.

**Figure 19:**
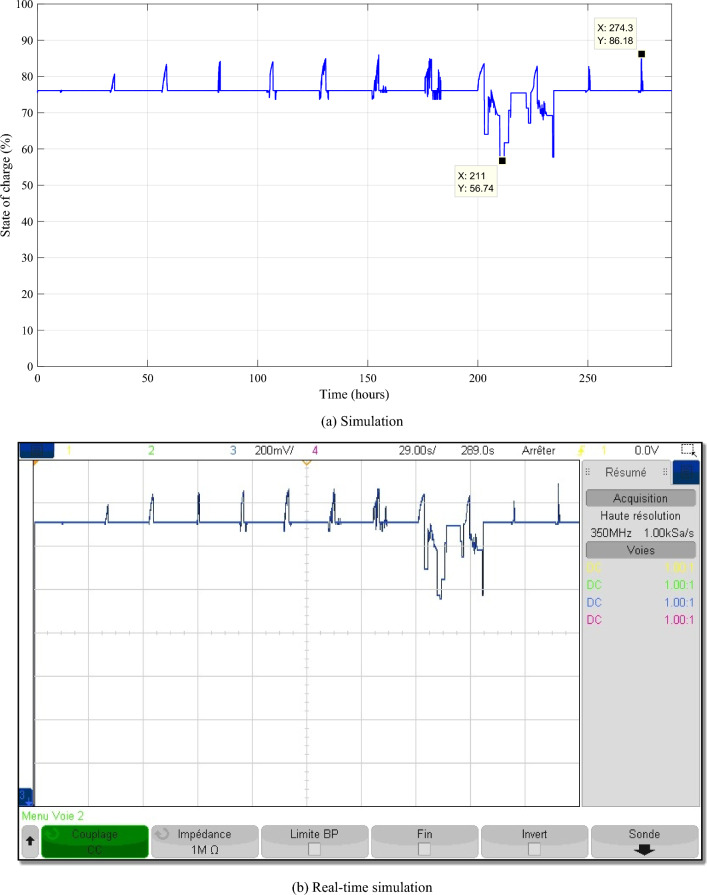
Battery state of charge.

It is noticed in Fig. 19, battery SOC is well controlled and is maintained between 56.74 and 86.18%. The batteries SOCs are kept within bounds, regardless of the variations in PV, wind and load power profiles.

**Figure 20:**
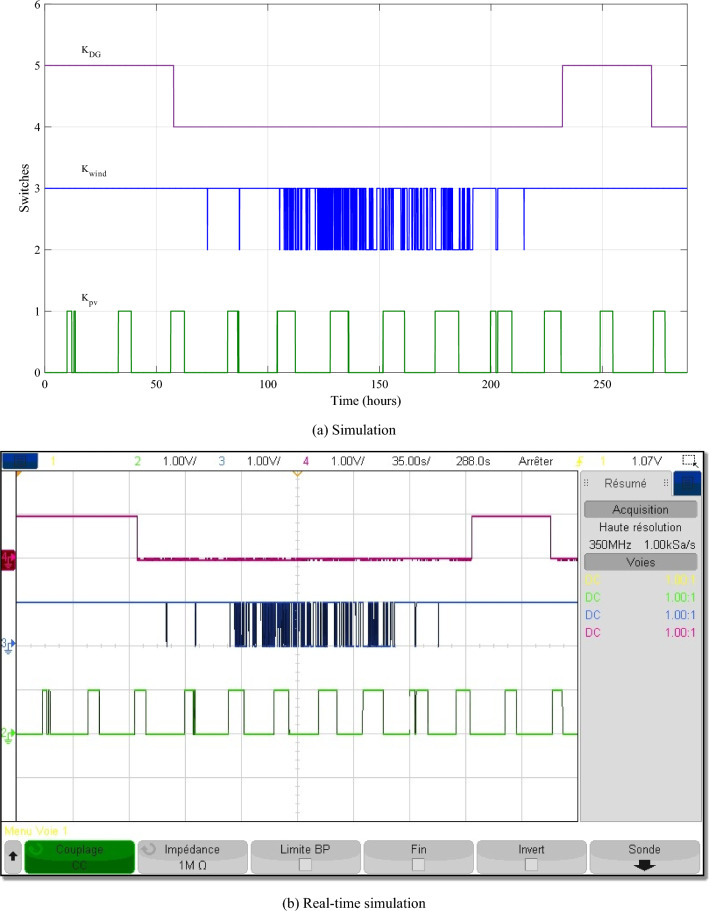
The different control signals.

The different control signals generated by the IPMC with FLC are given in the Fig. 20.

**Figure 21:**
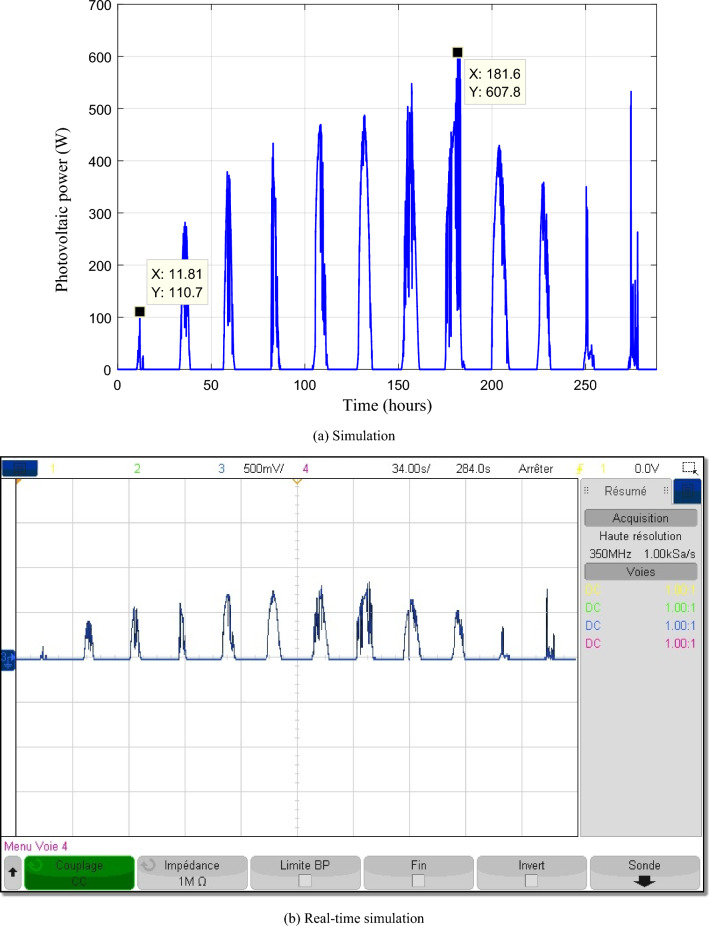
Photovoltaic power.

**Figure 22:**
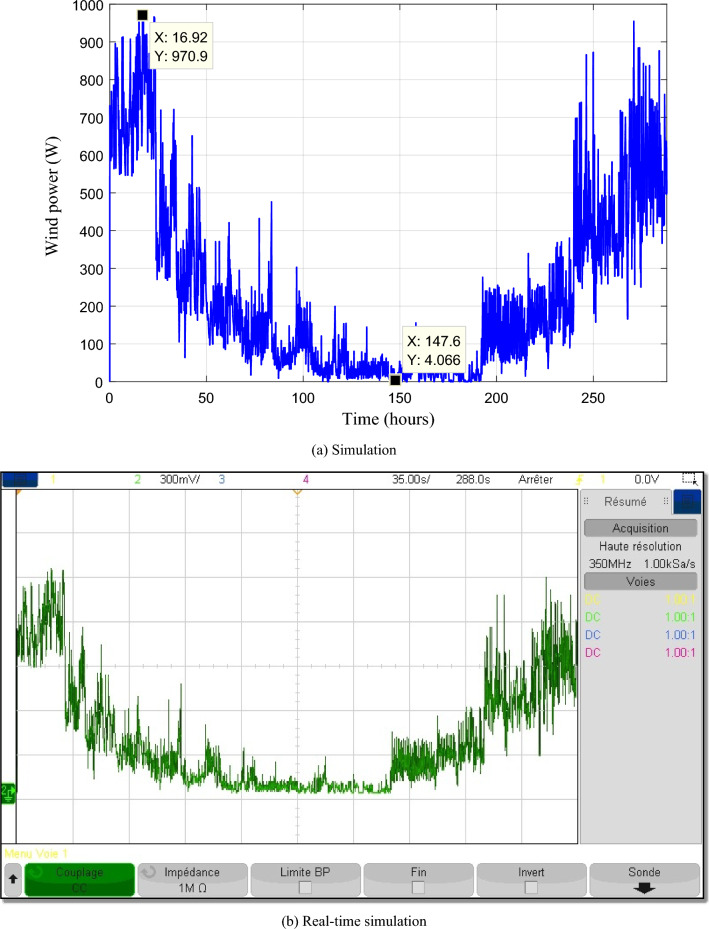
Wind turbine power.

Figures 21 and 22 depict respectively the PV and wind powers during the twelve profiles. The PV power varies from 110.7 to 607.80 W while the wind power varies from 4.066 to 970.90 W.

**Figure 23:**
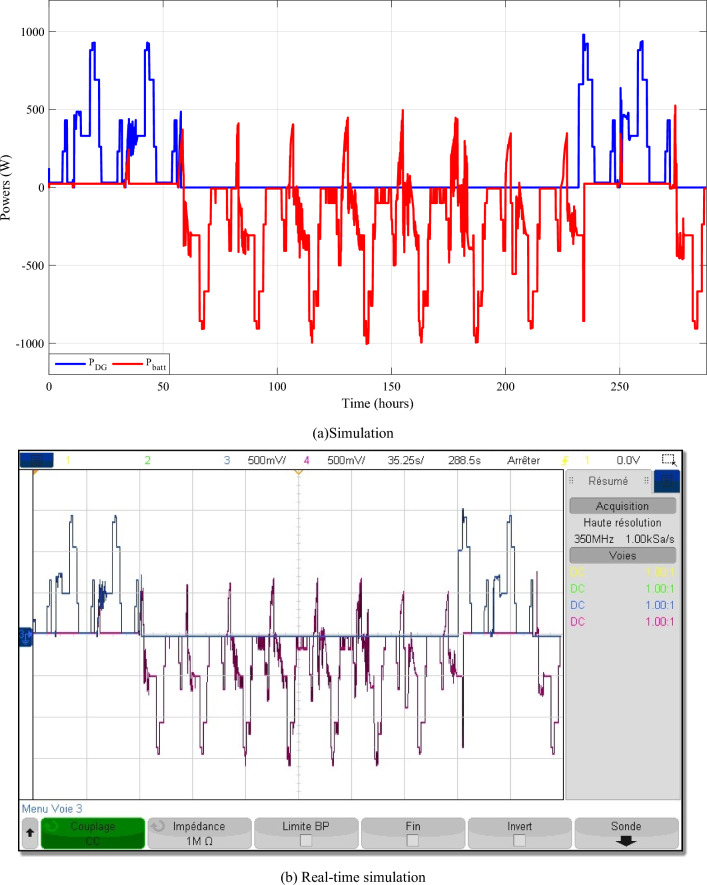
Diesel generator and batteries powers.

Batteries and DG powers are represented in the same curve (Fig. 23) to show that the DG only starts when the batteries are discharged, i.e. when the battery power is zero.

**Figure 24:**
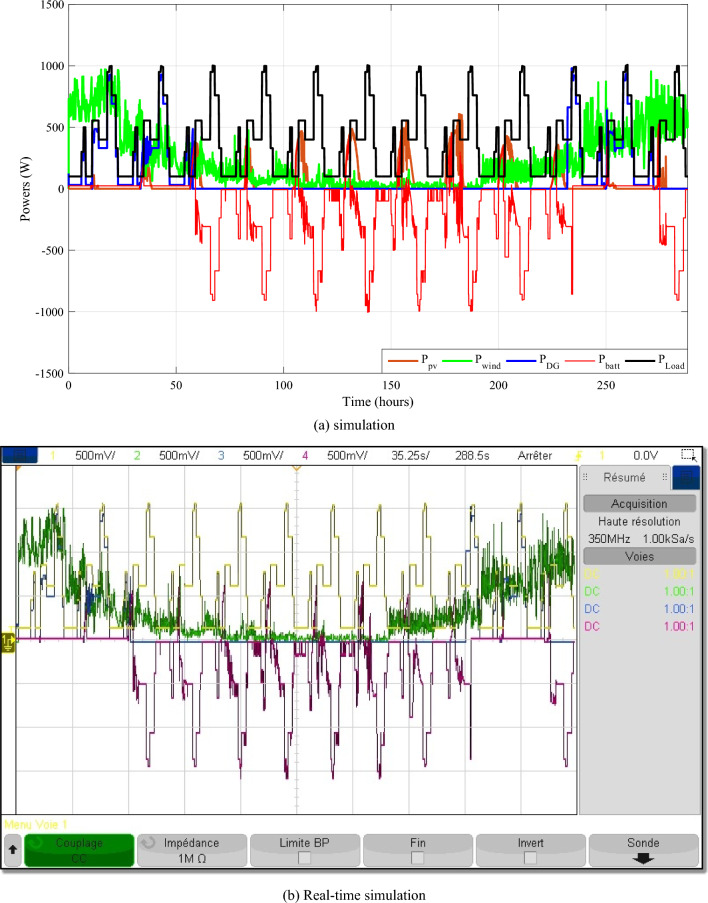
The different powers variation during twelve various profiles.

This scenario depicts a system of energy sources that relies on wind, solar, batteries, and a backup generator to provide dependable power. The system is meticulously designed to minimize generator utilization, instead relying on renewable sources, wind and solar, when available, and reserving the generator primarily for battery charging when required. This strategic approach serves to optimize energy consumption, reduce fuel consumption, and extend battery life. The power waveforms of the various sources are depicted in Fig. 24. Based on these findings, the proposed IPMC fulfills the load power need regardless of weather conditions.

**Figure 25:**
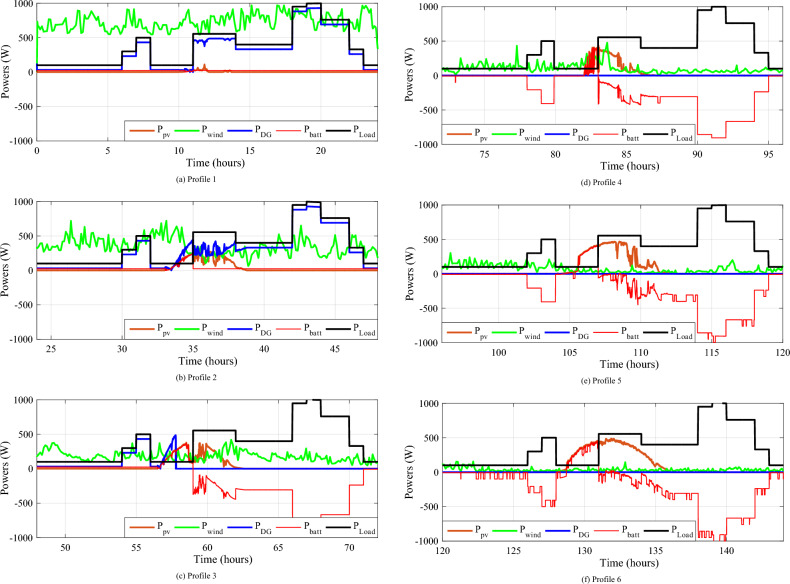

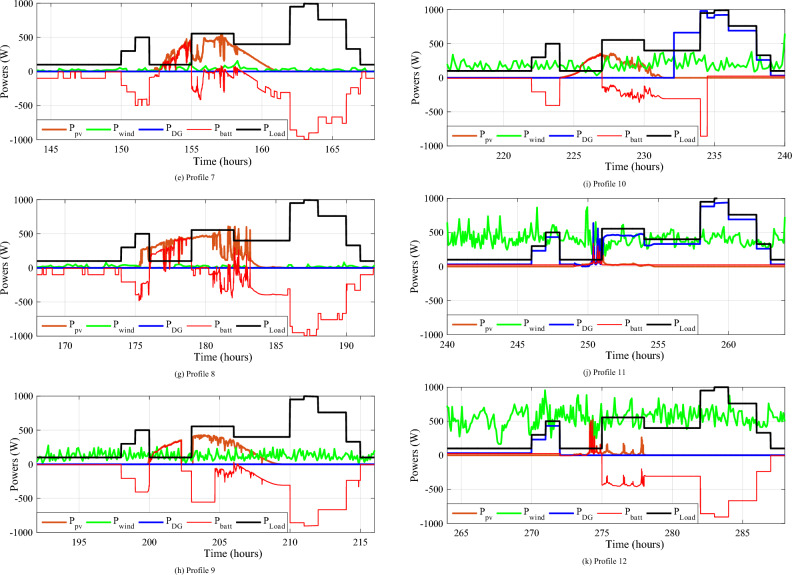
Zooms on the different powers-simulation.

Figure 25 displays the total power consumed each day by all power sources for twelve different profiles. The PV power changes with solar irradiation profile.

To better depict discharges in relation to PV, wind and load changes, battery powers are presented in negative. It should be noted that the negative sign of the batteries' powers indicates that they are supplying power, while the positive sign indicates that they are been charged. At start of operations, the batteries are not fully charged, and though wind energy production is substantial, it falls short of meeting the load requirements, prompting the DG to activate and provide power (Profile 1t o 3). Notably, over the course of six consecutive profiles (Profiles 4 to 9), solar irradiance remains consistently at an average of approximately 500 W/m^2^. During this phase, batteries recharge during daylight hours and provide compensation when solar irradiance levels decrease. In Profile 10, the batteries become depleted, necessitating the DG to take over load supply, as wind power is no longer a significant contributor. In the final phase, during profiles 11 to 12, increased wind speeds and average solar irradiance levels facilitate battery charging and compensation using both photovoltaic and wind power sources and DG to supply the load. It is clear that the DG was only used during the battery charging phase, with the twin goal of protecting the batteries and extending their operational life. It may be inferred that the load power was satisfactory over the different twelve average profiles throughout a year, owing to accurate sizing and, in part, to the proposed IPMC. It is clear that the power discharge represents just a modest quantity (negative regions are highlighted in red). Notably, the simulation results closely match those of the real-time simulation.

**Figure 26:**
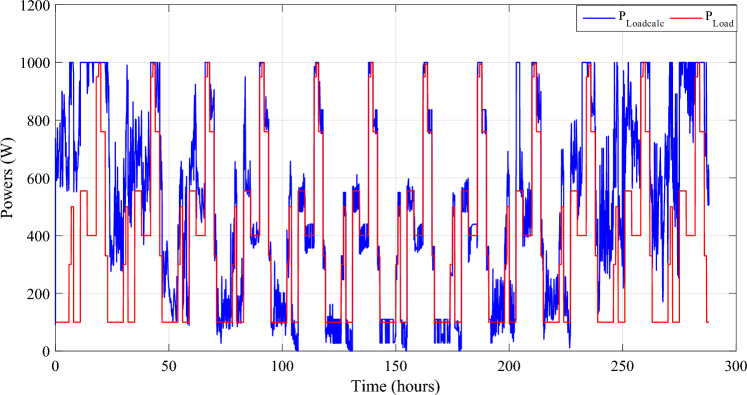
Calculated ${{\text{P}}}_{{\text{Loadcalc}}}$ and developed load power ${{\text{P}}}_{{\text{Load}}}$.

The reference load power and the sum of power developed by all the power sources are respectively shown in Fig. 26.

**Figure 27:**
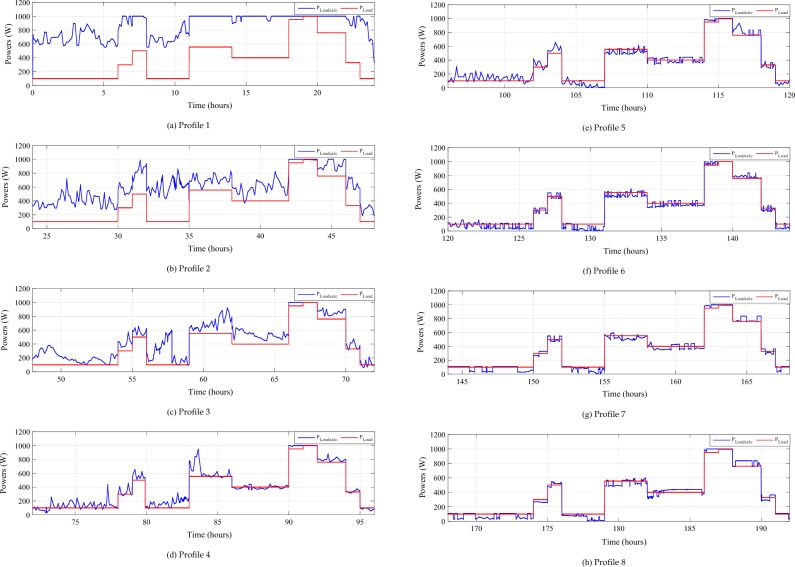

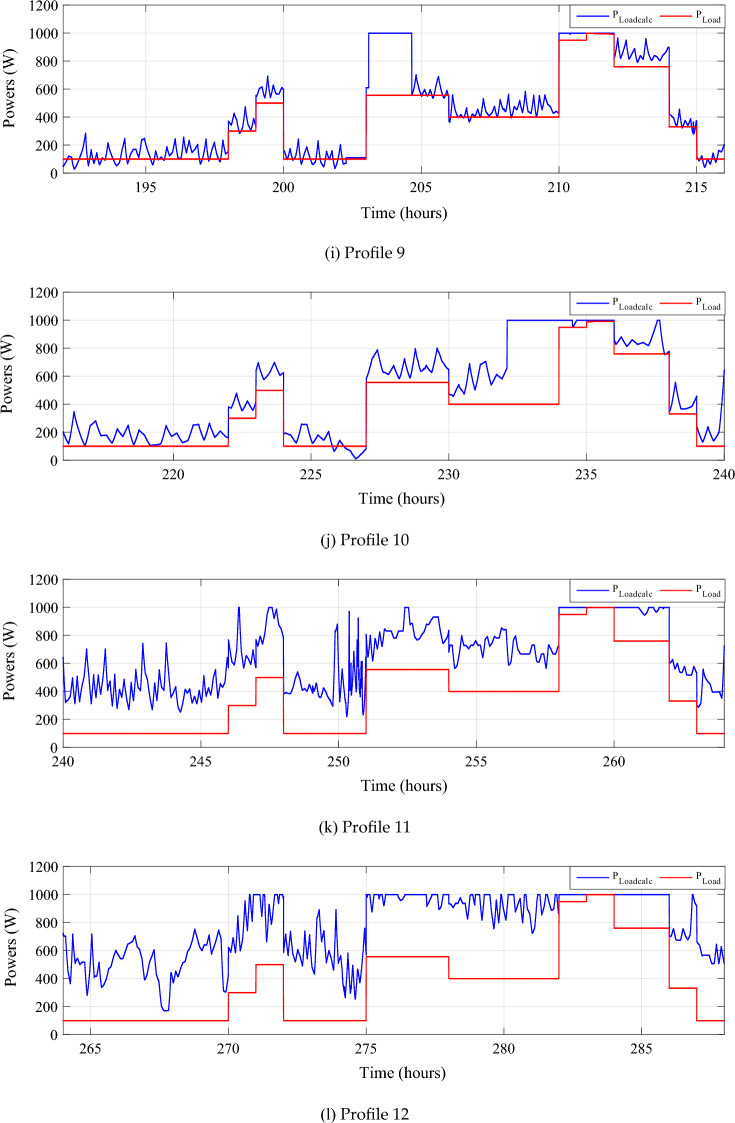
Zooms on calculated and developed load power.

The zoom of this last-mentioned figure for four distinct days is shown in Fig. 27.

In some cases, the computed power exceeds the power generated by the load. This surplus power is depicted in Fig. 27. Notably, even with adequate system sizing and the utilization of a Power Management Controller (PMC), a slight power surplus can be observed, during days of intense wind speeds and solar irradiance.

These visual illustrations serve to demonstrate the effectiveness of the proposed control and energy management methodology in terms of state of charge, current profiles, operational modes, power generation and consumption, as well as alignment with load requirements. These graphical representations offer valuable insights into the system's performance under various conditions, confirming the viability and practicality of the research approach.

## Conclusion

The study presents a promising approach to managing an autonomous hybrid energy system with a fuzzy logic controller. The novelty of the proposed IPMC lies in its dynamic and adaptive nature, leveraging fuzzy logic control to efficiently balance multiple power sources. Unlike traditional strategies, this approach considers variations in climatic conditions, contributing to improved system resilience. The dual-goal approach, emphasizing immediate power needs and long-term stability, adds a unique dimension compared to existing methods. Simulation results indicate that the proposed IPMC is effective. It successfully maintains power availability and keeps the battery at an optimal charge state. The study involves a comparison between real-time results obtained using an RT-LAB simulator and simulation results from MATLAB/Simulink. The results confirm the effectiveness and feasibility of the suggested control strategy at different profiles throughout a year. The main contribution of this work can be summarized as:

1. We have highlighted a research gap related to intelligent power management control (IPMC) for hybrid renewable energy systems (HRES). This sets the stage for the main contributions of the study.
2. A proposed innovative IPMC, utilizing fuzzy logic control (FLC), has been introduced as a novel approach to address the challenges in HRES. This introduces a new dimension to existing strategies, emphasizing the need for adaptive and dynamic control mechanisms.
3. The work recognizes the crucial need for a backup energy source in HRES due to the unreliability of solar irradiance and wind speed. The proposed IPMC ensures continuous power supply during unpredictable conditions, reducing dependence on conventional fossil fuels and enhancing the stability and reliability of HRES.
4. We recognize ongoing efforts to minimize the reliance on non-renewable backup sources by improving energy storage technologies and grid management. The proposed IPMC aligns with these efforts by providing an alternative strategy to optimize energy distribution in HRES.
5. The study employs MATLAB/Simulink simulations and real-time findings from the RT-LAB simulator, providing a comprehensive evaluation of the proposed IPMC. This adds a layer of validity and practical applicability to the contributions of the study.
6. The main contribution lies in the development of a dynamic and adaptive IPMC solution tailored to the specific challenges faced by HRES. Unlike existing strategies, the proposed IPMC considers variations in climatic conditions and efficiently balances multiple power sources, contributing to the overall resilience.
7. and reliability of HRES.
8. The work introduces a dual-goal approach of meeting immediate power needs and ensuring long-term stability and reliability. This emphasizes the holistic nature of the proposed IPMC, addressing both short-term and long-term objectives in HRES.
9. The study contributes to the advancement of intelligent power management strategies for sustainable and efficient energy systems. By introducing a new control mechanism, the research aims to enhance the overall performance and reliability of HRES.

To further advance this research and contribute to the practical implementation of such systems, some future research directions are planned as using adaptive control strategies and conduct a comprehensive techno-economic analysis to evaluate the cost-effectiveness of the proposed system compared to traditional energy sources.

## Data Availability

The datasets used and/or analysed during the current study available from the corresponding author on reasonable request.
